# Supplementary material for: Alphaherpesvirus pUL21 homologs use non-canonical sequences to compete with cellular adaptors for protein phosphatase 1 binding
Source: J Biol Chem. 2025 Nov 13;302(1):110936. doi: 10.1016/j.jbc.2025.110936 (PMC12723162; doi:10.1016/j.jbc.2025.110936)
Supplement: Supplementary figures [file mmc1.docx]

**
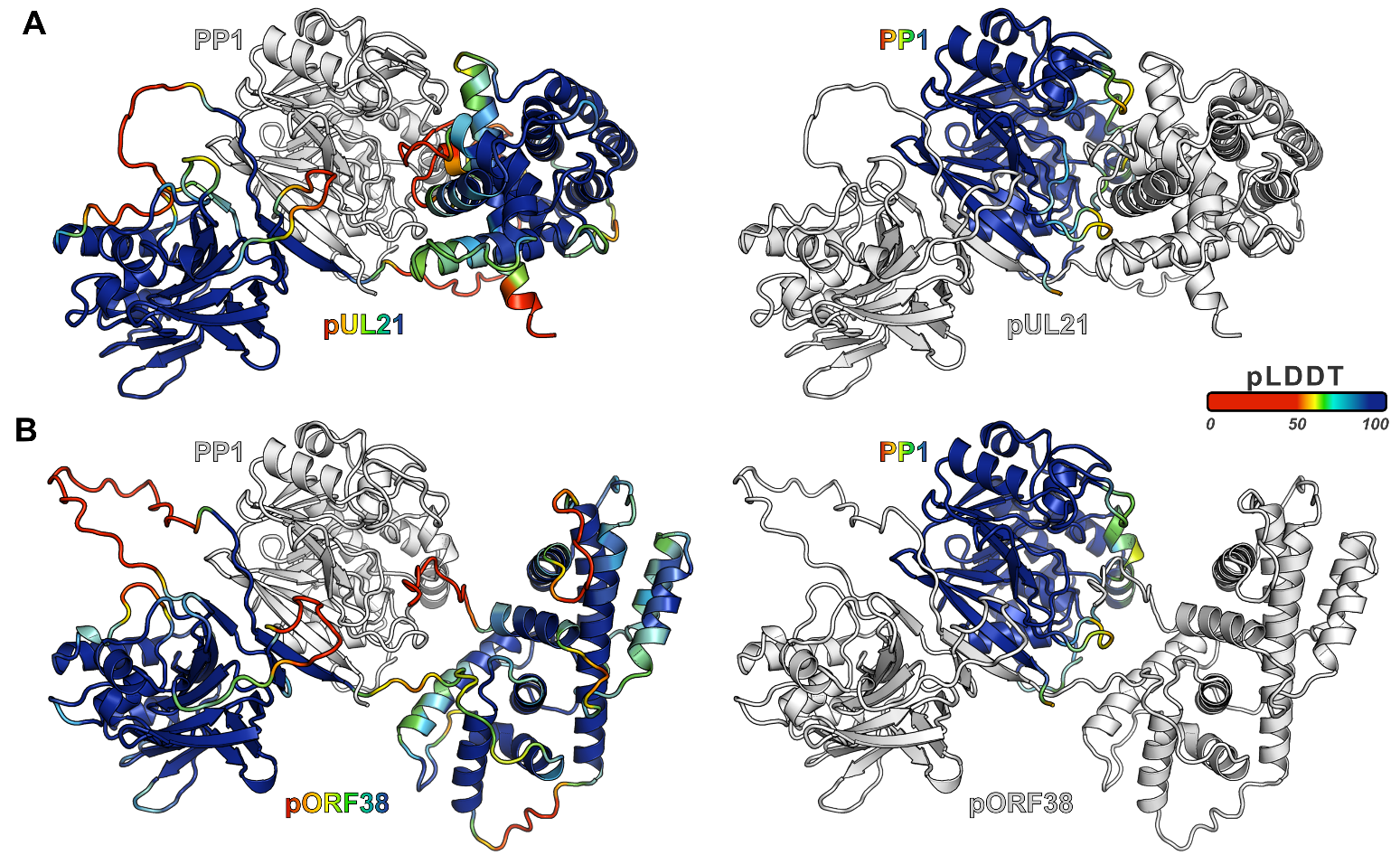
**

**Figure S1. Confidence of predicted complexes between PP1 and alphaherpesvirus pUL21 homologues.**

**A,B** AlphaFold2-Multimer models of human PP1γ catalytic domain (residues 7–300) in complex with **A** HSV-1 pUL21 or **B** VZV pORF38. Ribbons are coloured grey or by the predicted local distance difference test (pLDDT) scores as a spectrum from red (low confidence) to blue (high confidence). *Left* shows pUL21 homologue pLDDT scores and *Right* shows PP1 pLDDT scores.

**
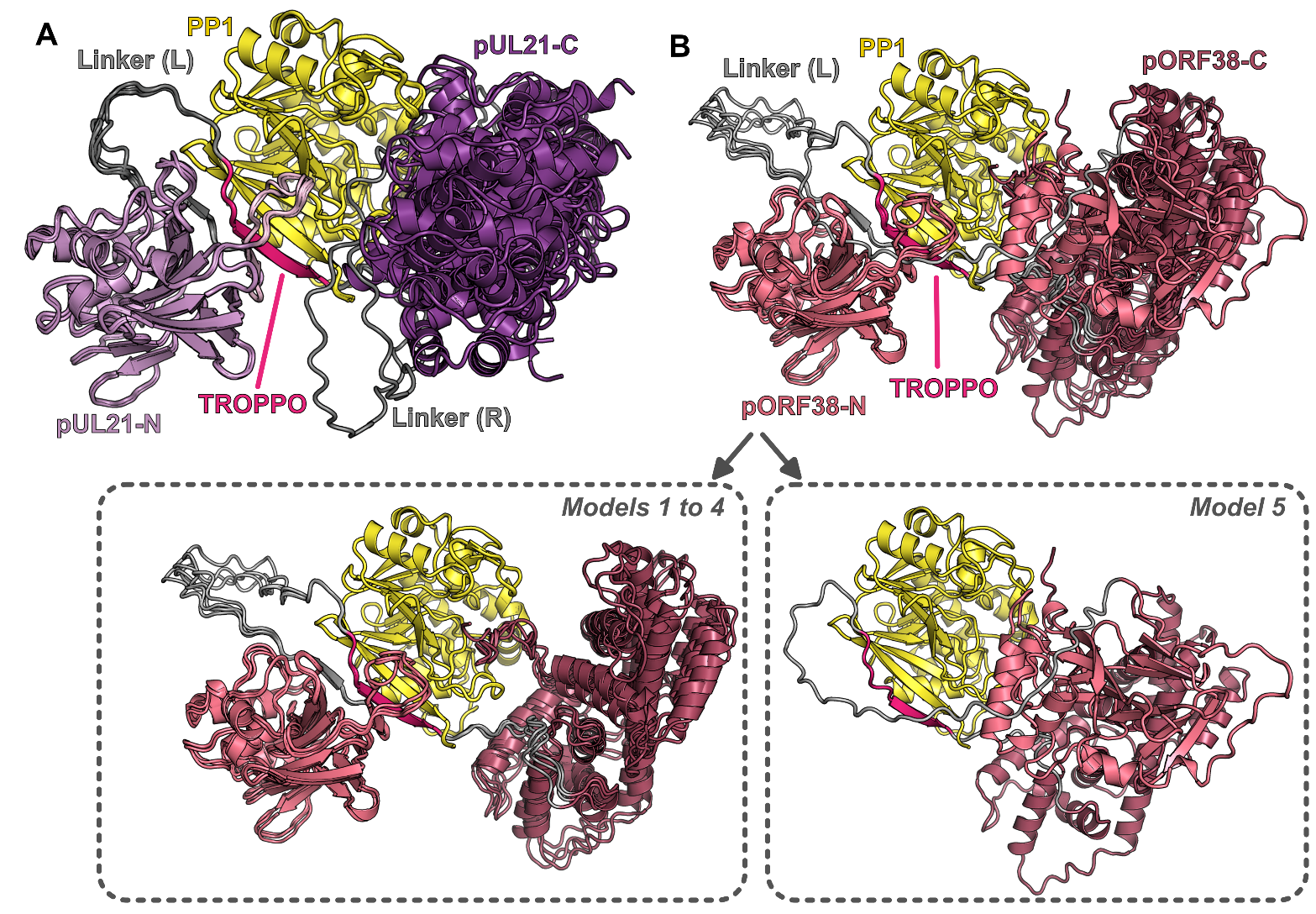
**

**Figure S2. Top five models for each predicted complex between alphaherpesvirus pUL21 homologues and PP1.**

**A,B** The five top scoring models of human PP1γ catalytic domain (residues 7–300; yellow ribbons) in complex with **A** HSV-1 pUL21 or **B** VZV pORF38 predicted by AlphaFold2-Multimer. Models are shown superposed via alignment of the PP1 catalytic domain and the pUL21 homologues are coloured by domain as in Fig. 3. The N-terminal domain and TROPPO motif of all five pUL21 models superpose well. There is variability in the orientation of the C-terminal domain with respect to PP1, consistent with the PAE matrix shown in Fig. 3D. Both the N- and C-terminal domains plus the TROPPO motif superpose well for four of the five models of pORF38 in complex with PP1, again consistent with the PAE matrix shown in Fig. 3D. For the fifth model the N-terminal domain adopts a different orientation, but the TROPPO motif still associates with the final β-strand of the PP1 catalytic domain to extend the PP1 β-sheet.


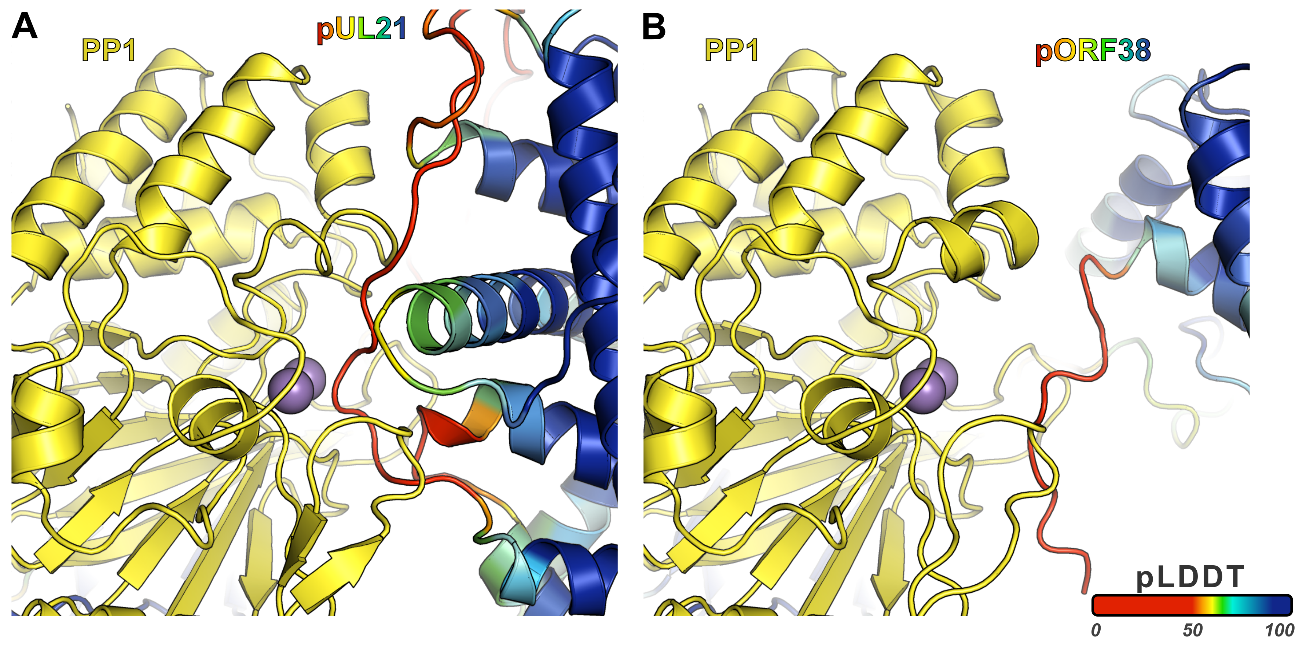


**Figure S3. Low confidence placement of alphaherpesvirus pUL21 homologue residues near the active site of PP1.**

**A,B** AlphaFold2-Multimer models of human PP1γ catalytic domain (residues 7–300; yellow ribbons) in complex with **A** HSV-1 pUL21 or **B** VZV pORF38 coloured by pLDDT scores from red (low confidence) to blue (high confidence). The position of the PP1 active site is denoted by the two Mn atoms (violet spheres) from a superposition of the crystal structure of PP1α catalytic domain in complex with the GADD34 RVxF+ϕϕ[xF] motif (PDB 4XPN) (28). Given their low confidence prediction, the positioning of these residues adjacent to the PP1 active site in the models is unlikely to have any functional relevance.


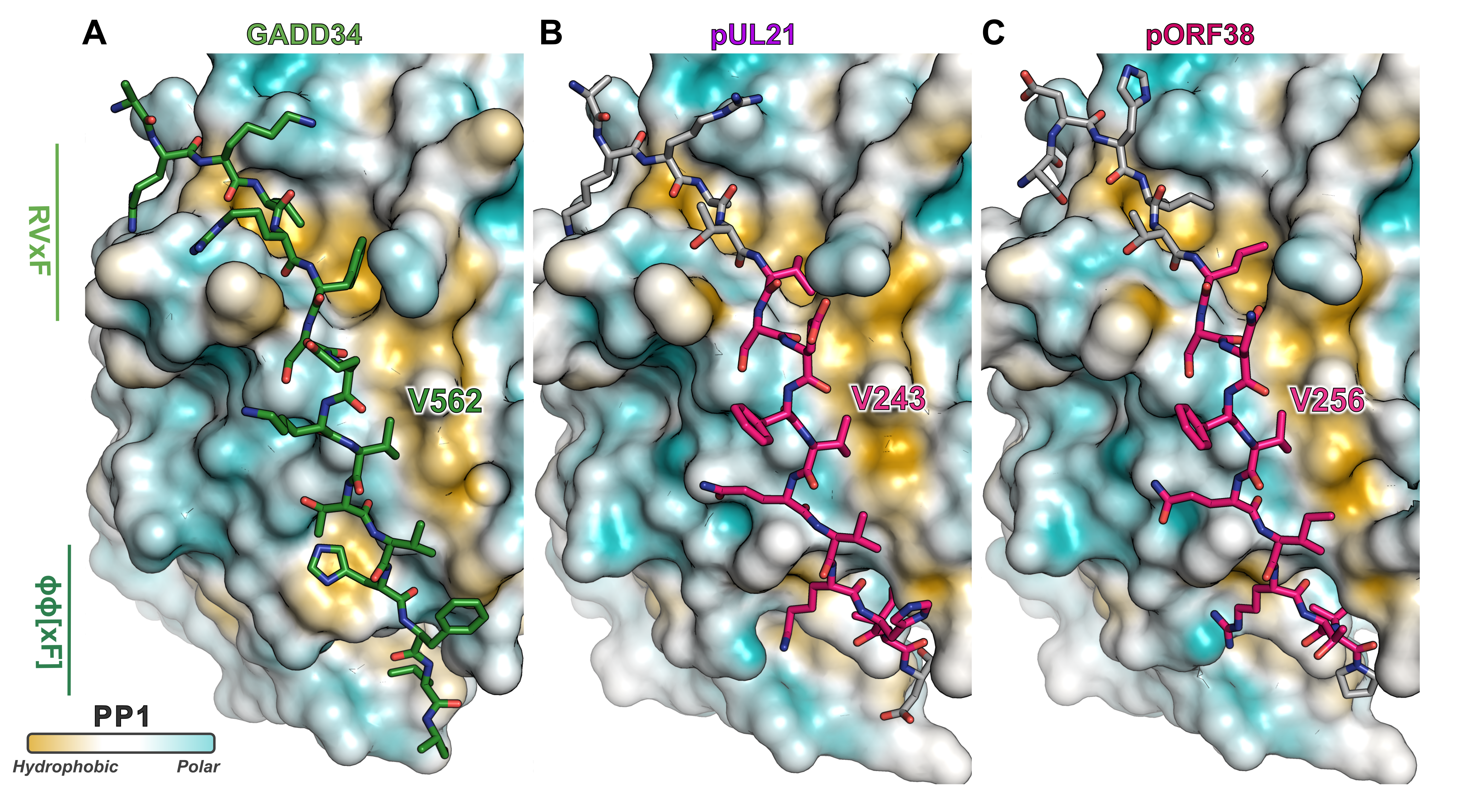
**Figure S4. Comparison of (predicted) peptide conformations at the PP1 hydrophobic groove.**

**A–C** PP1 is shown as a molecular surface coloured by mean lipophilicity, from orange (hydrophobic) to cyan (polar). **A** Structure of the crystal structure of PP1α catalytic domain in complex with the GADD34 RVxF+ϕϕ[xF] motif (PDB 4XPN) (28). The GADD34 peptide is shown as sticks with green carbon atoms. **B** AlphaFold2-Multimer model of PP1γ catalytic domain in complex with HSV-1 pUL21. The TROPPO motif (pink carbon atoms) and flanking residues (grey alpha carbons) are shown as sticks. **C** AlphaFold2-Multimer model of PP1γ catalytic domain in complex with VZV pORF38. The TROPPO motif (pink carbon atoms) and flanking residues (grey alpha carbons) are shown as sticks.


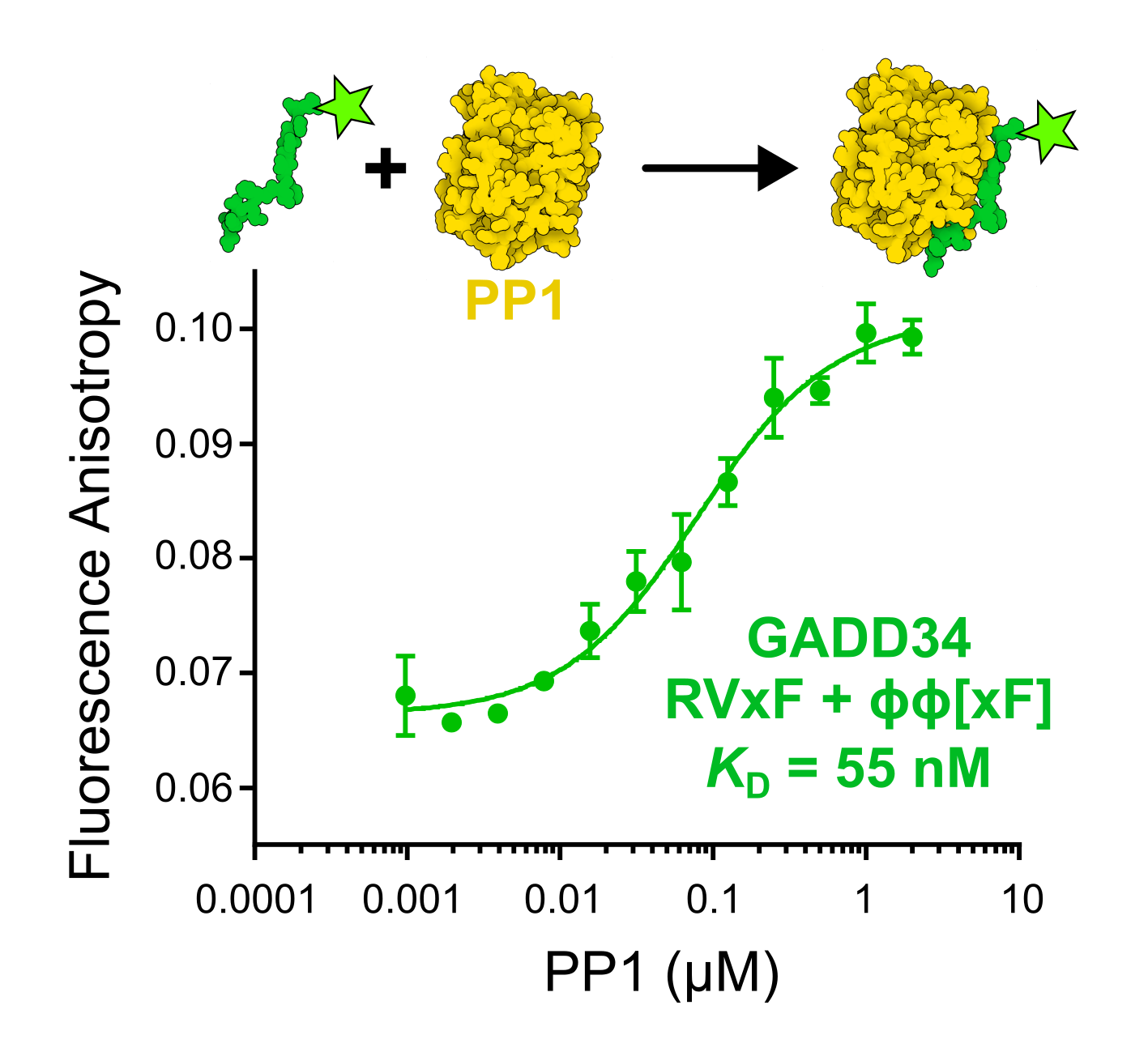


**Figure S5. Association of GADD34 peptide and PP1 in Tris pH 8.5 buffer.**

Fluorescence anisotropy of PP1γ catalytic domain (residues 7–300) binding 2 nM fluorescently labelled peptide containing the GADD34 RVxF and ϕϕ[xF] motifs (residues 552–568). Mean ± SD is shown for technical triplicate measurements. Affinity (*K*_D_) is average of three independent experiments.
