# Supplementary material for: Alphaherpesvirus pUL21 homologs use non-canonical sequences to compete with cellular adaptors for protein phosphatase 1 binding
Source: J Biol Chem. 2025 Nov 13;302(1):110936. doi: 10.1016/j.jbc.2025.110936 (PMC12723162; doi:10.1016/j.jbc.2025.110936)
Supplement: Table S1 [file mmc2.docx]

**Table S1.** **Isothermal titration calorimetry (ITC) of PP1 with TROPPO-containing peptides and proteins.** Data for independent experiments are shown. For all, the cell contained C-terminally His_6_-tagged mouse PP1γ (7–300). –, no binding detected.

| **Titrant** | **[Titrant] in syringe (µM)** | **[PP1] in cell (µM)** | ***K*_D_ (nM)** | **∆H (kcal/mol)** | **∆G (kcal/mol)** | **-T∆S (kcal/mol)** | **N (sites)** |
| --- | --- | --- | --- | --- | --- | --- | --- |
| pUL21-TROPPO (234–250) | 1000 | 58.8 | – | – | – | – | – |
|  | 1000 | 54.5 | – | – | – | – | – |
| pORF38-TROPPO (246–263) | 1000 | 79.6 | – | – | – | – | – |
|  | 500 | 17.0 | – | – | – | – | – |
|  | 500 | 16.0 | – | – | – | – | – |
| pORF38-NLT-His_6_ (1–263) | 240 | 17.0 | 719 | -13.1 | -8.38 | 4.76 | 1.21 |
|  | 147 | 10.0 | 540 | -13.3 | -8.55 | 4.72 | 0.965 |
|  | 153 | 9.5 | 231 | -13.6 | -9.06 | 4.5 | 0.838 |
